# Trypsinogen isoforms in the ferret pancreas

**DOI:** 10.1038/s41598-018-33423-w

**Published:** 2018-10-10

**Authors:** Eszter Hegyi, Miklós Sahin-Tóth

**Affiliations:** Center for Exocrine Disorders, Department of Molecular and Cell Biology, Boston University Henry M. Goldman School of Dental Medicine, Boston, MA 02118 USA

## Abstract

The domestic ferret (*Mustela putorius furo*) recently emerged as a novel model for human pancreatic diseases. To investigate whether the ferret would be appropriate to study hereditary pancreatitis associated with increased trypsinogen autoactivation, we purified and cloned the trypsinogen isoforms from the ferret pancreas and studied their functional properties. We found two highly expressed isoforms, anionic and cationic trypsinogen. When compared to human cationic trypsinogen (PRSS1), ferret anionic trypsinogen autoactivated only in the presence of high calcium concentrations but not in millimolar calcium, which prevails in the secretory pathway. Ferret cationic trypsinogen was completely defective in autoactivation under all conditions tested. However, both isoforms were readily activated by enteropeptidase and cathepsin B. We conclude that ferret trypsinogens do not autoactivate as their human paralogs and cannot be used to model the effects of trypsinogen mutations associated with human hereditary pancreatitis. Intra-pancreatic trypsinogen activation by cathepsin B can occur in ferrets, which might trigger pancreatitis even in the absence of trypsinogen autoactivation.

## Introduction

The digestive protease precursor trypsinogen is synthesized and secreted by the pancreas to the duodenum where it becomes activated to trypsin^1^. The activation process involves limited proteolysis of the trypsinogen activation peptide by enteropeptidase, a brush-border serine protease specialized for this sole purpose. The activation peptide is typically an eight amino-acid long N-terminal sequence, which contains a characteristic tetra-aspartate motif preceding the activation site peptide bond, which corresponds to Lys23-Ile24 in human trypsinogens. Active trypsin participates in the digestion of dietary proteins in the gut and serves as the specific activator of all other digestive protease precursors, i.e. chymotrypsinogens, proelastases and procarboxypeptidases. Trypsin can also activate its own precursor by cleaving the trypsinogen activation peptide at Lys23. This process, termed trypsinogen autoactivation, can result in the ectopic activation of trypsinogen in the pancreas, where it can lead to the inflammatory disorder pancreatitis^2^. In animal models of experimentally induced pancreatitis, trypsinogen becomes activated in the acinar cells by the lysosomal cysteine protease cathepsin B^3^. Human genetic studies and biochemical analyses of mutant trypsinogen forms provided convincing evidence for the pathogenic role of trypsinogen autoactivation in pancreatitis^2^, whereas the role of cathepsin B in human pancreatitis remains unclear^4^. In humans, the digestive protease chymotrypsin C (CTRC) regulates autoactivation of cationic trypsinogen by cleaving regulatory nick sites; at Leu81 in the calcium-binding loop and at Phe18 in the activation peptide^2,5^. Cleavage after Leu81 results in protective trypsinogen degradation facilitated by a trypsin-mediated autolytic cleavage of the Arg122-Val123 peptide bond. Cleavage after Phe18, on the other hand, stimulates autoactivation as the shortened activation peptide becomes a better substrate for trypsin. Under physiological conditions, trypsinogen degradation is the prevailing effect of CTRC, which protects the pancreas against pancreatitis. Trypsinogen mutations associated with hereditary pancreatitis can render trypsinogen resistant to CTRC-mediated degradation and/or increase processing of the activation peptide resulting in accelerated autoactivation and higher intra-pancreatic trypsin levels^2,5^.

To understand the role of trypsinogen autoactivation in the development of pancreatitis, animal models that mimic the human disease both phenotypically and mechanistically are required. Genetic modification of animal trypsinogens is complicated by the fact that mammals express a variable number of highly similar isoforms. Thus, the human pancreas produces two major isoforms, cationic trypsinogen (PRSS1, serine protease 1) and anionic trypsinogen (PRSS2), and the minor isoform mesotrypsinogen (PRSS3). Even though the advent of genomics provided us with trypsinogen sequences for a large number of animals; genomic predictions cannot identify which isoforms are expressed to high levels and how these isoforms autoactivate. Therefore, classical biochemical and cloning approaches remain essential to characterize trypsinogens in potential model animals. In this regard, previously we purified and cloned guinea pig trypsinogen and found a single isoform, which was completely defective in autoactivation, indicating that the guinea pig would be a poor model to study hereditary pancreatitis^6^. On the other hand, purification of trypsinogens from the resting mouse pancreas revealed four abundantly expressed isoforms and we identified the mouse cationic trypsinogen (isoform 7) as a promising model for human PRSS1^7^. The domestic ferret recently emerged as a novel model animal for the studies of pancreatic diseases^8,9^. Genetic deletion of the cystic fibrosis transmembrane conductance regulator (CFTR) resulted in cystic fibrosis with features typical of the human disease, including rapidly progressive chronic pancreatitis with eventual pancreatic insufficiency. Here, we set out to purify and clone ferret trypsinogens to evaluate whether these would be appropriate models for human hereditary pancreatitis driven by trypsinogen autoactivation.

## Materials and Methods

### Animals

Flash-frozen ferret pancreas from adult female ferrets (*Mustela putorius furo*, sable coat color) was a kind gift from John Engelhardt (University of Iowa). The pancreata were shipped on dry ice and stored at −80 °C until use.

### Accession codes

The ferret pre-trypsinogen cDNA sequences have been deposited to GenBank under accession numbers MH499474, MH499475, MH499476, MH499477, and MH499478.

### Purification of ferret trypsinogens

Pancreatic tissue (1,580 mg) was homogenized in 3 mL of 0.1 M Tris-HCl (pH 8.0) using a rotor-stator tissue homogenizer (IKA T25 Basic S1) at 13,000 rpm and a tissue grinder (mortar size 5 mL). The homogenate was centrifuged for 5 min at 13,200 rpm, at 5 °C. The supernatant (2.5 mL) was further clarified by a second centrifugation, diluted 1:1 with 0.1 M Tris-HCl (pH 8.0) and subjected to a third centrifugation. Approximately 1 mL of the third supernatant was loaded onto a 2 mL ecotin-affinity column equilibrated with 0.1 M Tris-HCl (pH 8.0) and 0.2 M NaCl. The ecotin column was washed with 20 mM Tris-HCl (pH 8.0), 0.2 M NaCl and elution was carried out with 50 mM HCl. Five mL eluate was collected. Four mL of the ecotin column eluate was directly loaded onto a Mono S column equilibrated with 20 mM Na-acetate (pH 5.0). The column was developed with a 0–0.5 M NaCl gradient (in 20 mM Na-acetate, pH 5.0) at 1 mL/min flow rate. Peak fractions were collected and analyzed by SDS-PAGE and enzyme activity assays.

### Complementary DNA cloning

Total RNA was isolated from 47 mg ferret pancreas using the RNeasy Plus Mini Kit (Qiagen, Valencia, CA). Reverse transcription and 5′ and 3′ RACE reactions were carried out with the SMARTer RACE 5′/3′ Kit (catalog #634858, Clontech, Mountain View, CA) with 1 µg RNA. As gene-specific primers for the initial RACE reactions, we used sense and antisense primers, which anneal to the region around the conserved catalytic Ser200 codon (in bold underlined type). Sense primer, 5′-TGT CAG GGA GAC **TCT** GGT GGC CCA GTT GTC TGC-3′, antisense primer, 5′-GCA GAC AAC TGG GCC ACC **AGA** GTC TCC CTG-3′. These primers were originally designed against the corresponding region of the *Bothrops jararaca* trypsinogen (GenBank AF190273) and used previously for the cloning of the guinea pig trypsinogen^6^. PCR products were purified and directly subcloned into the pCR4-TOPO TA vector using the TOPO TA Cloning Kit for Sequencing (catalog #K457501, Life Technologies, Carlsbad CA). Based on the initial sequencing results from the TOPO clones, new trypsinogen-specific primers were designed and used to amplify the different isoforms. PCR products were sequenced directly. Primer sequences are listed in Supplementary Figs S1–S3. To investigate linkage of two variants found in ferret cationic trypsinogen, an additional 3′ RACE reaction was performed using the forward gene-specific primer. PCR products were TOPO-cloned and sequenced.

### Expression plasmids

The three ferret trypsinogen isoforms were PCR-amplified from pancreatic cDNA and cloned into the pTrapT7 trypsinogen expression plasmid using NcoI and SalI restriction sites^10^. In these constructs, the signal peptide of the pre-trypsinogen was replaced with a Met-Ala sequence, which results in cytoplasmic expression in *E. coli*. The primers used for cloning the ferret trypsinogens were as follows; the restriction sites are shown in bold underlined type. Anionic sense, 5′-TTT AAA T**CC ATG G**CT TTC CCC ACT GAT GAG GAT GAC AAG ATC-3′, Anionic antisense, 5′-AAA TTT **GTC GAC** TTA GCT GTT GGC AGC TAT GGT CGT CTT AAT-3′; Cationic sense, 5′-TTT AAA T**CC ATG G**CT TTC CCC ATT GAT GAC GAT GAC AAG ATC −3′, Cationic antisense, 5′-AAA TTT **GTC GAC** TTA GTT GGC AGA AAT GGT TTG CCG AAT CCA-3′; Minor sense, 5′-TTT AAA T**CC ATG G**CT GTC CCC ATT GAG GAT GAT GAC AAG ATC-3′, Minor antisense, 5′-AAA TTT **GTC GAC** TTA GTT GGC AGC AAT GGT CTC CTG AAT CCA-3′.

### Expression and purification of recombinant trypsinogen

Ferret trypsinogens and human cationic trypsinogen were expressed in *Escherichia coli* BL21(DE3) as cytoplasmic inclusion bodies. Expression, *in vitro* refolding and purification with ecotin-affinity chromatography followed our standard protocols originally developed for human trypsinogens^10–12^. Concentrations of trypsinogen solutions were determined from the UV absorbance at 280 nm using the following theoretical extinction coefficients, PRSS1 37,525 M^−1^ cm^−1^, ferret anionic trypsinogen, 41,660 M^−1^ cm^−1^, ferret cationic trypsinogen, 37,650 M^−1^ cm^−1^.

### Trypsinogen activation with enteropeptidase

Ferret trypsinogens and human cationic trypsinogen (PRSS1) were incubated at 2 µM concentration with 28 ng/mL human enteropeptidase (catalog #1585-SE, R&D Systems, Minneapolis, MN) at 37 °C, in 0.1 M Tris-HCl (pH 8.0), 1 mM CaCl_2_ and 0.05% Tween 20 (final concentrations) in a final volume of 100 µL. Aliquots of 2 µL were withdrawn from reaction mixtures at the indicated times and trypsin activity was determined with the synthetic substrate *N*-CBZ-Gly-Pro-Arg-*p*-nitroanilide.

### Trypsinogen autoactivation experiments

Ferret trypsinogens and human cationic trypsinogen (PRSS1) were incubated at 37 °C in 0.1 M Tris-HCl (pH 8.0) and 0.05% Tween 20 in 1 mM or 10 mM CaCl_2_, with or without 10 nM initial trypsin (final concentrations), as indicated in the figure legends. Aliquots (2 µL) were withdrawn at given times and trypsin activity was measured with the *N*-CBZ-Gly-Pro-Arg-*p*-nitroanilide peptide substrate.

### Trypsinogen activation with cathepsin B

Ferret trypsinogens were activated at 1 µM concentration with 8 µg/mL human cathepsin B (catalog #219362, Calbiochem) at 37 °C in 0.1 M sodium acetate buffer (pH 4.0), 1 mM K-EDTA and 0.05% Tween 20, in 100 µL final volume. Cathepsin B was incubated with 1 mM dithiothreitol (DTT) before use; the final carryover concentration of DTT in the activation mixture was 20 µM. Aliquots (2 µL) were withdrawn at indicated times and trypsin activity was measured with the *N*-CBZ-Gly-Pro-Arg-*p*-nitroanilide substrate.

### Kinetic analysis

Michaelis-Menten parameters of ferret trypsins were determined in 0.1 M Tris-HCl (pH 8.0), 1 mM CaCl_2_, and 0.05% Tween 20 at 22 °C. Increasing concentrations (0 to 160 μM) of the *N*-CBZ-Gly-Pro-Arg-*p*-nitroanilide peptide substrate were incubated with 1 nM trypsin and initial velocities of substrate cleavage were plotted as a function of the substrate concentration. Data points were fitted with the Michaelis-Menten hyperbolic equation.

### Protease activity measurements

Trypsin and chymotrypsin activity were assayed at 22 °C using the synthetic chromogenic substrates *N*-CBZ-Gly-Pro-Arg-*p*-nitroanilide and *Suc*-Ala-Ala-Pro-Phe-*p*-nitroanilide, respectively. The substrates were dissolved at 200 µM concentration in assay buffer (0.1 M Tris-HCl (pH 8.0), 1 mM CaCl_2_, 0.05% Tween 20). Samples were diluted to 50 µL with assay buffer and the cleavage reaction was started by adding 150 µL substrate. Release of the yellow *p*-nitroanilin was followed for 1 min at 405 nm in a microplate reader. Rates of substrate cleavage were determined from the initial linear portions of the curves and activity was expressed in mOD/min units.

### SDS-PAGE analysis

Proteins were precipitated with 10% trichloroacetic acid (final concentration); pelleted with centrifugation and dissolved in 20 µL Laemmli sample buffer containing 100 mM DTT. After heat-denaturation for 5 min at 95 °C, samples were electrophoresed on 15% minigels and stained with Coomassie Blue.

### Quantitative real-time PCR

Absolute expression levels of trypsinogen isoforms were determined using SYBR Green-based real-time quantitative PCR with the following primers: Anionic forward, 5′-AGC TGT CCT CGC CTG CTG TC-3′, Anionic reverse, 5-GGT AGT TGA TGC CAG AGC TC-3′; Cationic forward, 5′-AAC TGA GCT CTC CCG CCA CC-3′, Cationic reverse, 5-GAT ACC TTT GCC CAG TAC TC-3′; Minor forward, 5′-CCA GAT ACA ATG AAC AAA ACT TTG-3′, Minor reverse, 5′-CTG GGT ATC AAC ATC CGC AC-3′. Product sizes were 129 bp (anionic and cationic trypsinogens) and 125 bp (minor trypsinogen). Standard curves were generated using 10-fold serial dilutions of plasmids harboring the coding DNA for anionic, cationic and minor ferret trypsinogen. Samples were analyzed in triplicates.

## RESULTS

### Genomic sequence predicts three ferret trypsinogen isoforms

The genome assembly of a female ferret (MusPutFur1.0) was released on June 2, 2011^13^, and this sequence was used to predict trypsinogens in subsequent annotations in 2013 and 2015 (Table 1). Three trypsinogen isoforms were predicted, two anionic and one cationic.

**Table 1:** Trypsinogen isoforms predicted from the ferret genome sequence (NW_004569535.1).

Accession	Gene	Name	pI	Date	Match with cDNA cloning
XM_004781428.1	LOC101683959	anionic trypsin-like	5.03	June 13, 2013	Anionic trypsinogen
XM_004781428.2	LOC101683959	anionic trypsin	5.03	July 1, 2015	Anionic trypsinogen
*XM_004814591.1*	*LOC101683959*	*anionic trypsin-like*	*5.03*	*June 13, 2013*	*Anionic trypsinogen*
XM_004781430.1	LOC101684544	cationic trypsin-like	8.71	June 13, 2013	Cationic trypsinogen
XM_004781430.2	LOC101684544	cationic trypsin	8.71	July 1, 2015	Cationic trypsinogen
*XM_004814585.1*	*LOC101670962*	*cationic trypsin-like*	*5.29*	*June 13, 2013*	*Minor trypsinogen*
XM_004782606.1	LOC101670962	cationic trypsin-like partial	n/a	June 13, 2013	Minor trypsinogen
XM_004782606.2	LOC101670962	cationic trypsin-like partial	n/a	July 1, 2015	Minor trypsinogen

The NCBI records created on June 13, 2013 were either updated or removed (shown in italics) on July 1, 2015. pI, theoretical isoelectric point of trypsinogen. See text for more details.

### Purification of trypsinogen isoforms from the ferret pancreas

**Figure 1:**
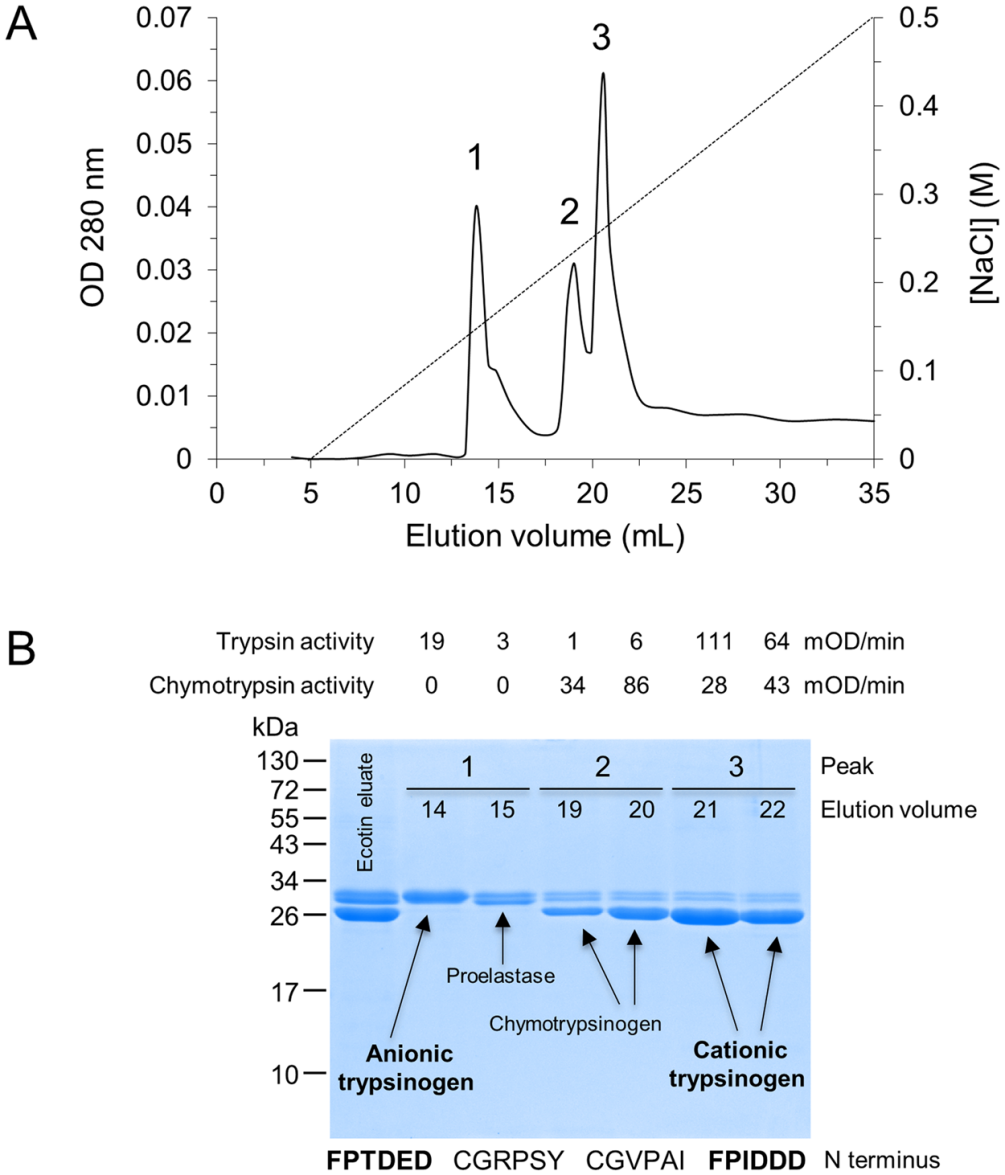
Purification of ferret trypsinogen isoforms. (**A**) Mono S ion-exchange chromatography of ferret trypsinogens. Pancreas homogenate was pre-purified on an ecotin affinity column, which binds trypsinogens and to a lesser degree chymotrypsinogens and proelastases. The ecotin eluate was loaded onto the Mono S column and proteins were eluted with a 0–0.5 M gradient of NaCl at a flow rate of 1 mL/min. (**B**) SDS-PAGE analysis of the ecotin-eluate (50 µL of 5 mL eluate loaded) and Mono S peaks 1, 2 and 3 (180 µL from 1 mL fraction loaded). See *Methods* for details. Trypsin and chymotrypsin activity of the fractions were also measured, as described in *Methods*. N-terminal protein sequencing was used to identify major protein bands; the first five residues were determined.

**Figure 2:**
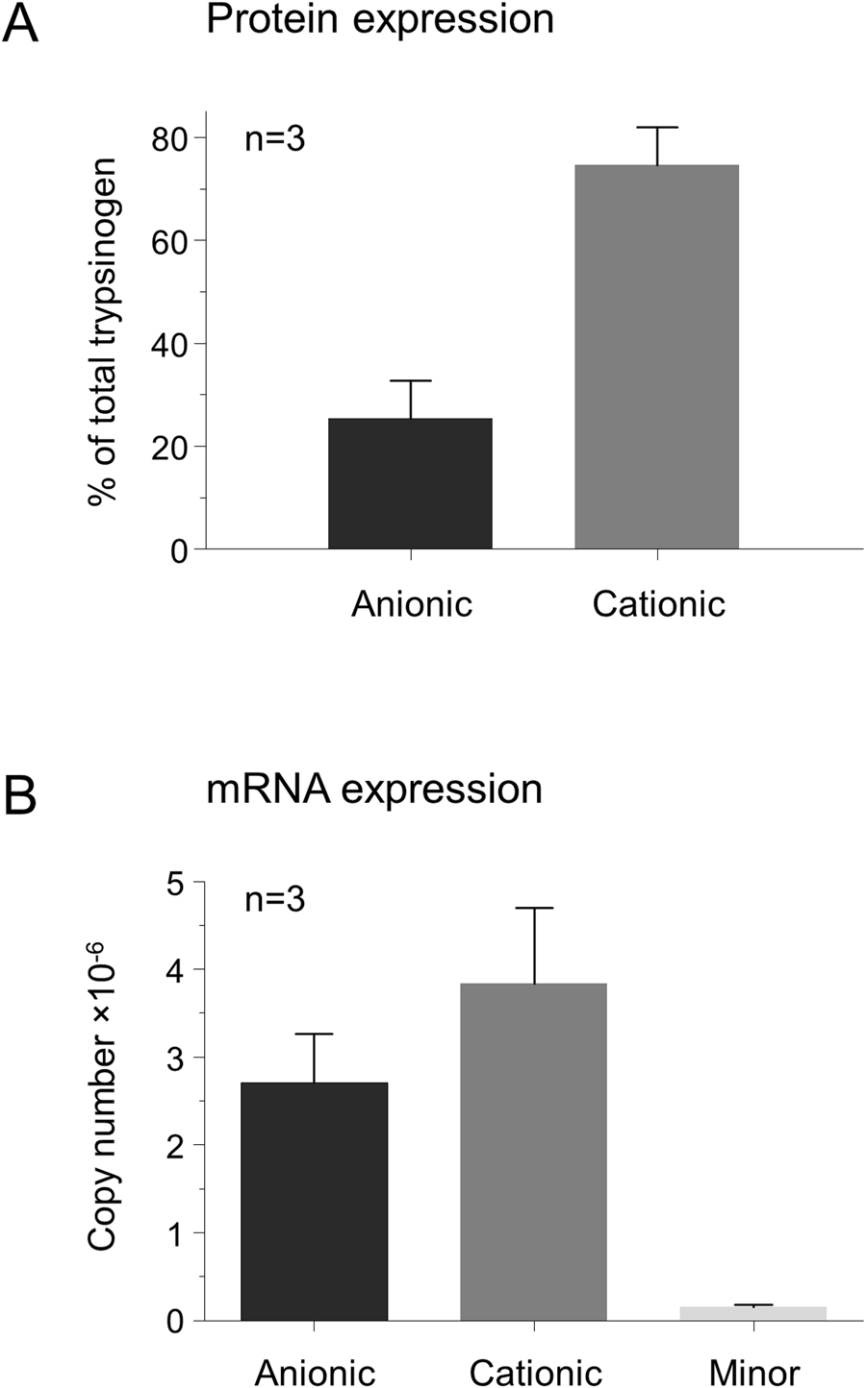
Expression levels of trypsinogen isoforms in the ferret pancreas. Mean values with S.D. are shown. (**A**) Protein expression was estimated from the trypsin activity of the Mono S chromatography peaks. (**B**) Expression of mRNA was measured by reverse transcription and quantitative real-time PCR, as detailed in *Methods*.

To identify and quantitate the trypsinogen isoforms expressed by the ferret pancreas, we followed the purification strategy we employed previously for the isolation of mouse and guinea pig trypsinogens^6,7^. This approach takes advantage of the fact that the pan-serine-protease inhibitor ecotin binds trypsinogen and ecotin-affinity chromatography can be used to capture trypsinogens from the pancreas homogenate. As detailed in *Methods*, we loaded clarified pancreatic homogenate onto an ecotin column, washed the column with Tris-HCl buffer (pH 8.0) and eluted trypsinogens with 50 mM HCl. The flow-through from the ecotin-column was devoid of trypsinogen, as judged by the lack of trypsin activity after activation with enteropeptidase. In contrast, the same assay yielded high trypsin activity in the eluate, indicating that we quantitatively isolated all trypsinogens from the ferret pancreas. In addition to trypsinogens, the ecotin eluate was expected to contain also some chymotrypsinogen and proelastase, as ecotin binds these serine protease zymogens as well. To resolve the different protein species in our crude trypsinogen preparation, we subjected the ecotin eluate to Mono S ion-exchange chromatography at pH 5.0 (Fig. 1A). The three major peaks that eluted from the Mono S column were analyzed by SDS-PAGE and Coomassie Blue staining and activity assays for trypsin and chymotrypsin (Fig. 1B). Finally, all major bands were transferred to PVDF membrane and their N-terminal amino-acid sequence was determined by Edman-degradation. Based on their enzymatic activity and N-terminal sequences, we identified two major trypsinogen isoforms; anionic trypsinogen in peak 1 and cationic trypsinogen in peak 3. The tail end of peak 1 also contained a proelastase and the major band in peak 2 corresponded to a chymotrypsinogen. We determined the relative abundance of the anionic and cationic trypsinogen isoforms from three independent purifications and found that cationic trypsinogen was expressed to higher levels, representing about 2/3 of total trypsinogen (Fig. 2A).

### Complementary DNA cloning of ferret trypsinogens

We prepared total RNA from the ferret pancreas and transcribed it to cDNA, as detailed in *Methods*. Using primers that anneal to a conserved region of the trypsinogen sequences around the catalytic Ser residue, we performed 5′ and 3′ RACE PCR reactions. The amplicons were cloned into the pCR4-TOPO TA vector and 20 clones from the 5′ RACE reaction and 15 clones from the 3′ RACE reaction were sequenced. The 5′ RACE clones revealed three distinct sequences, 14 clones matched genomic prediction XM_004781428.2, five clones matched XM_004781430.2 and one clone matched XM_004814585.1. The 3′ clones contained two different sequences matching XM_004781428.2 (five clones) and XM_004781430.2 (ten clones). Based on the sequences obtained, new gene-specific primers were designed annealing close to the 5′ and 3′ ends. Each isoform was then PCR-amplified from the pancreatic cDNA and the products were sequenced directly. These results unambiguously confirmed the three trypsinogen isoforms that matched the genomic predictions described above. Based on their isoelectric point (see Table 1) or abundance (i.e. number of RACE clones found) we designated these isoforms **anionic trypsinogen** (XM_004781428.2), **cationic trypsinogen** (XM_004781430.2) and **minor trypsinogen** (XM_004814585.1). DNA sequences and predicted amino-acid sequences are presented in Supplementary Figs S1–S3. While the anionic trypsinogen sequence we determined was a perfect match with genomic prediction XM_004781428.2, the cDNA cloning revealed two variants in cationic trypsinogen, the synonymous c.123T > G (p.L41=) variant and the 3′ UTR variant c.*35C > T. Since electropherogram peak heights for the heterozygous signals were comparable and genomic predictions did not identify a second cationic trypsinogen gene, the two variants likely represent linked allelic variants. We performed additional 3′ RACE reactions using the forward gene-specific primer for cationic trypsinogen followed by TOPO TA cloning and confirmed that the two variants were indeed linked. The sequence for the minor trypsinogen isoform differed from the genomic prediction at position c.651, where we found a homozygous C nucleotide instead of the predicted T. This change would represent a silent, synonymous variant, p.G217=. Furthermore, we found the allelic variant c.37G > A (p.A13T) which alters the minus 3 amino-acid in the signal peptidase recognition motif and thereby may affect secretion of the minor trypsinogen.

### mRNA expression levels of ferret trypsinogen isoforms

Using quantitative real-time PCR and appropriate plasmid standards, we determined the cDNA copy numbers for the three trypsinogen isoforms. As shown in Fig. 2B, only anionic and cationic trypsinogens were expressed to high levels, while the minor trypsinogen isoform was hardly detectable. In agreement with the expression data at the protein level (see Fig. 2A); there was more cationic trypsinogen expressed at the mRNA level as well, although the difference between the two isoforms was smaller.

### Functional properties of anionic and cationic ferret trypsinogens

**Figure 3:**
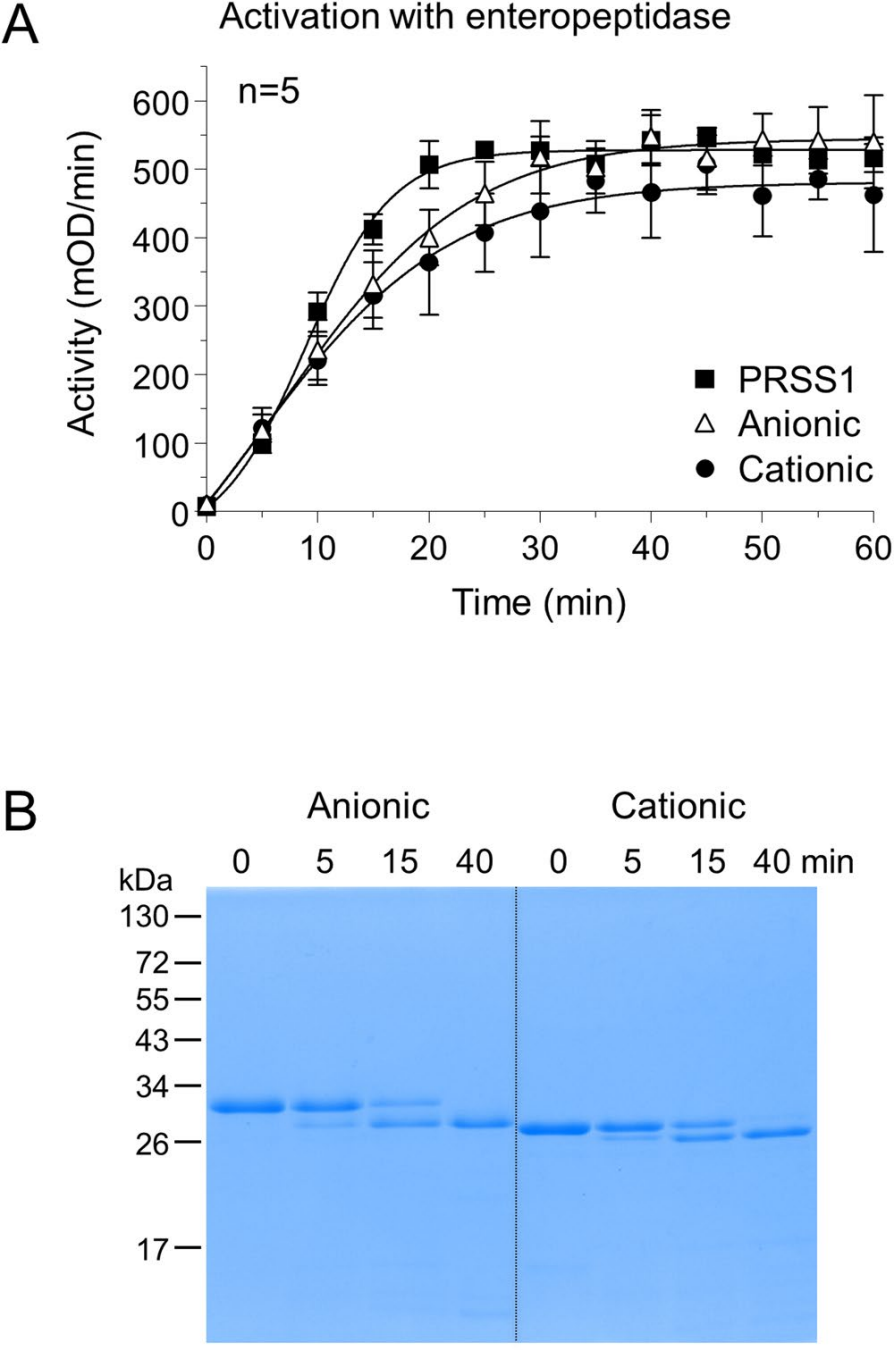
Activation of ferret trypsinogens and human cationic trypsinogen (PRSS1) by enteropeptidase. (**A**) Trypsinogens at 2 µM concentration were incubated with 28 ng/mL human enteropeptidase (final concentration) at 37 °C, in 0.1 M Tris-HCl (pH 8.0), 1 mM CaCl_2_ and 0.05% Tween 20 (final concentrations) in a final volume of 100 µL. Aliquots of 2 µL were withdrawn at the indicated times and trypsin activity was measured with the *N*-CBZ-Gly-Pro-Arg-*p*-nitroanilide substrate. Mean values with S.D. are shown. (**B**) Samples (90 µL) were precipitated with 10% trichloroacetic acid (final concentration) at the indicated times and analyzed by SDS-PAGE and Coomassie Blue staining, as described in *Methods*. A representative gel from two experiments is shown.

**Figure 4:**
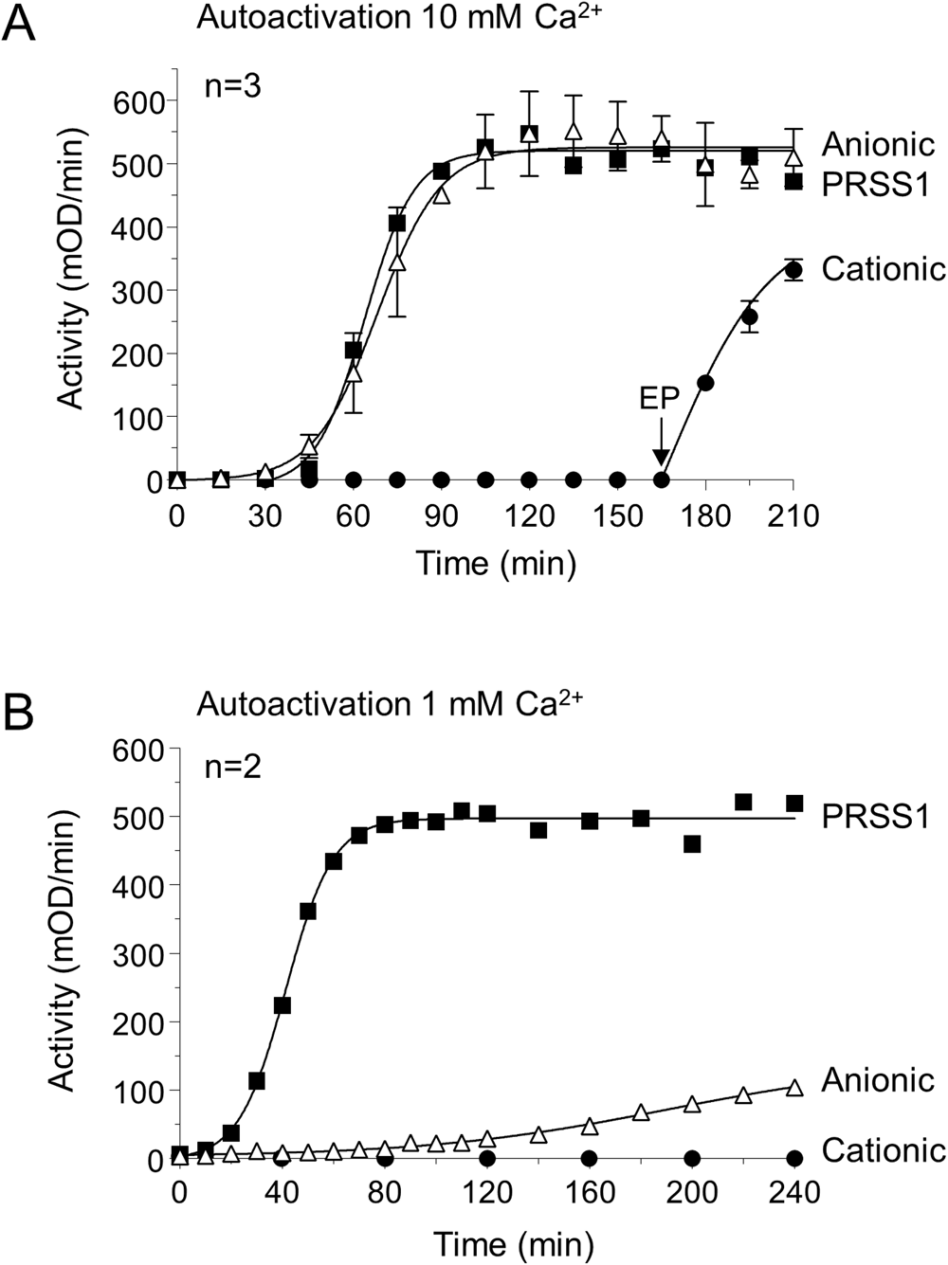
Autoactivation of ferret trypsinogens and human cationic trypsinogen (PRSS1). Trypsinogens (2 µM) were incubated at 37 °C, in 0.1 M Tris-HCl (pH 8.0), and 0.05% Tween 20 (final concentrations). Aliquots of 2 µL were withdrawn at the indicated times and trypsin activity was measured with the *N*-CBZ-Gly-Pro-Arg-*p*-nitroanilide substrate. (**A**) Autoactivation in the presence of 10 mM CaCl_2_, with no initial trypsin added. The arrow indicates addition of 28 ng/mL human enteropeptidase (EP). Mean values with S.D. are shown. (**B**) Autoactivation in the presence of 1 mM CaCl_2_, with 10 nM initial trypsin added. Mean values from two experiments are shown.

We recombinantly expressed and purified ferret anionic and cationic trypsinogen and compared their activation to human PRSS1. The minor trypsinogen isoform was not studied further. Activation with enteropeptidase showed very similar kinetics for anionic, cationic and human PRSS1 trypsinogens (Fig. 3A). SDS-PAGE analysis with Coomassie Blue staining confirmed quantitative activation with no degradation (Fig. 3B). Surprisingly, however, when autoactivation was measured under optimal catalytic conditions (pH 8.0, 10 mM calcium) only the anionic isoform activated similarly to human PRSS1 (Fig. 4A). Cationic ferret trypsinogen, in contrast, developed no trypsin activity over the time course of the experiment. Addition of enteropeptidase resulted in rapid activation, indicating that the preparation could become activated and no degradation occurred during incubation. We repeated the autoactivation experiment in 1 mM calcium, which best approximates physiological conditions in the secretory pathway (Fig. 4B). Under these conditions, ferret anionic trypsinogen exhibited very slow autoactivation while the cationic isoform was completely defective again. Thus, compared to human PRSS1, both ferret trypsinogens autoactivated poorly in 1 mM calcium.

**Figure 5:**
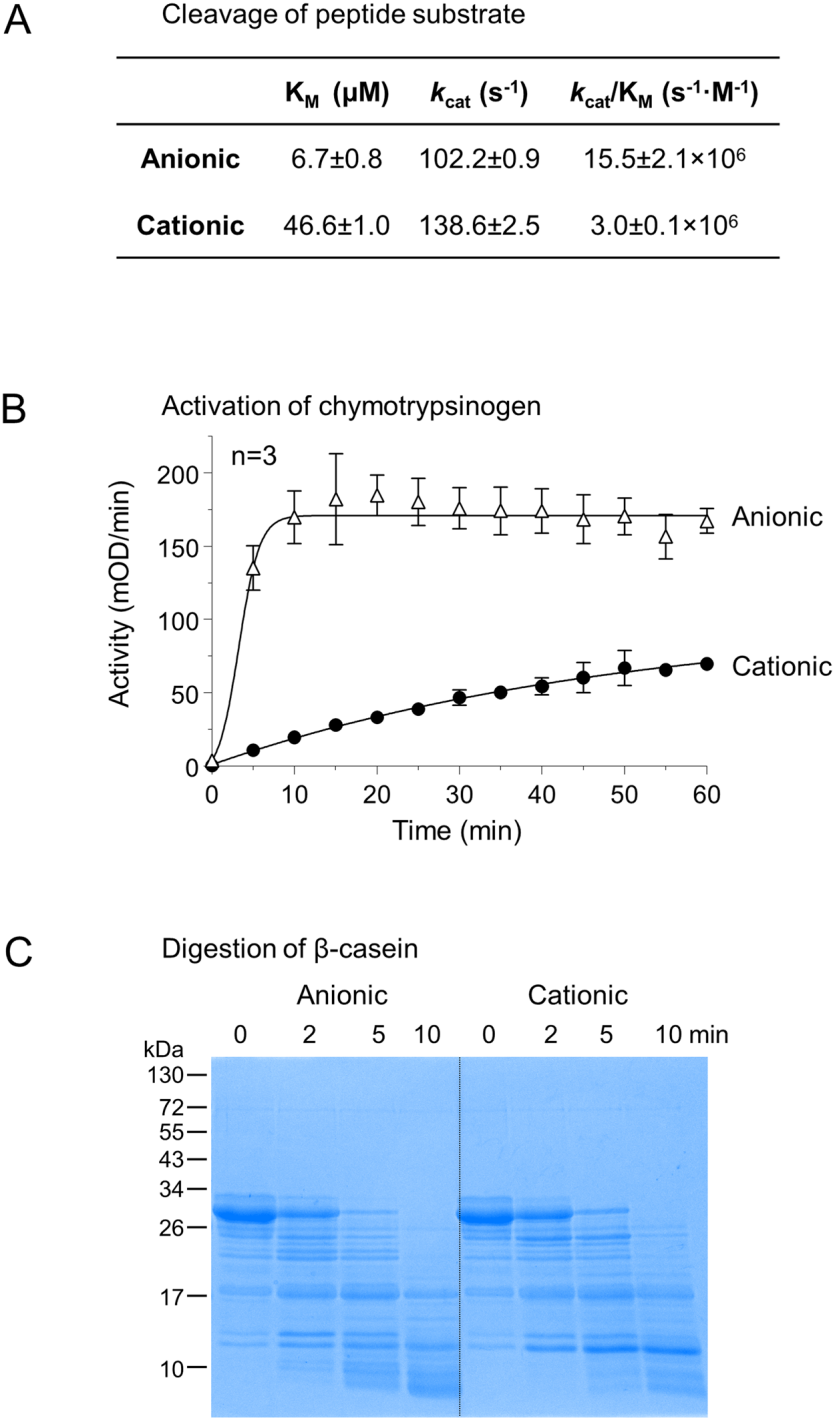
Catalytic activity of ferret anionic and cationic trypsins. (**A**) Enzyme kinetic parameters determined on the *N*-CBZ-Gly-Pro-Arg-*p*-nitroanilide substrate at 22 °C. Incubations were performed in 0.1 M Tris-HCl (pH 8.0), 1 mM CalCl_2_ and 0.05% Tween 20 with 1 nM trypsin (final concentrations). (**B**) Activation of bovine chymotrypsinogen (2 µM) with 25 nM ferret trypsins at 37 °C in 0.1 M Tris-HCl (pH 8.0), 1 mM CalCl_2_ and 0.05% Tween 20 (final concentrations). At the indicated times 2 µL aliquots were withdrawn and chymotrypsin activity was measured with the *Suc*-Ala-Ala-Pro-Phe-*p*-nitroanilide substrate. Mean values with S.D. are shown. (**C**) Digestion of bovine β-casein with ferret trypsins. Casein (0.2 mg/mL) was incubated with ferret trypsin (5 nM) in 0.1 M Tris·HCl (pH 8.0) and 1 mM CaCl_2_ (final concentrations) at 37 °C. At the indicated times, 75 µL aliquots were precipitated with 10% trichloroacetic acid, electrophoresed on 15% SDS-PAGE minigels, and stained with Coomassie Blue. A representative gel from two experiments is shown.

To establish whether the autoactivation was due to catalytic impairment, we measured Michaelis-Menten parameters on a small peptide substrate (Fig. 5A). Both trypsins exhibited high catalytic efficiency, although anionic trypsin was 5-times more active which was mainly due its 7-times lower K_M_ value. A similar (~10-fold) activity difference between the two isoforms was also evident in the chymotrypsinogen activation assay (Fig. 5B). In contrast, both isoforms digested β-casein at comparable rates (Fig. 5C).

**Figure 6:**
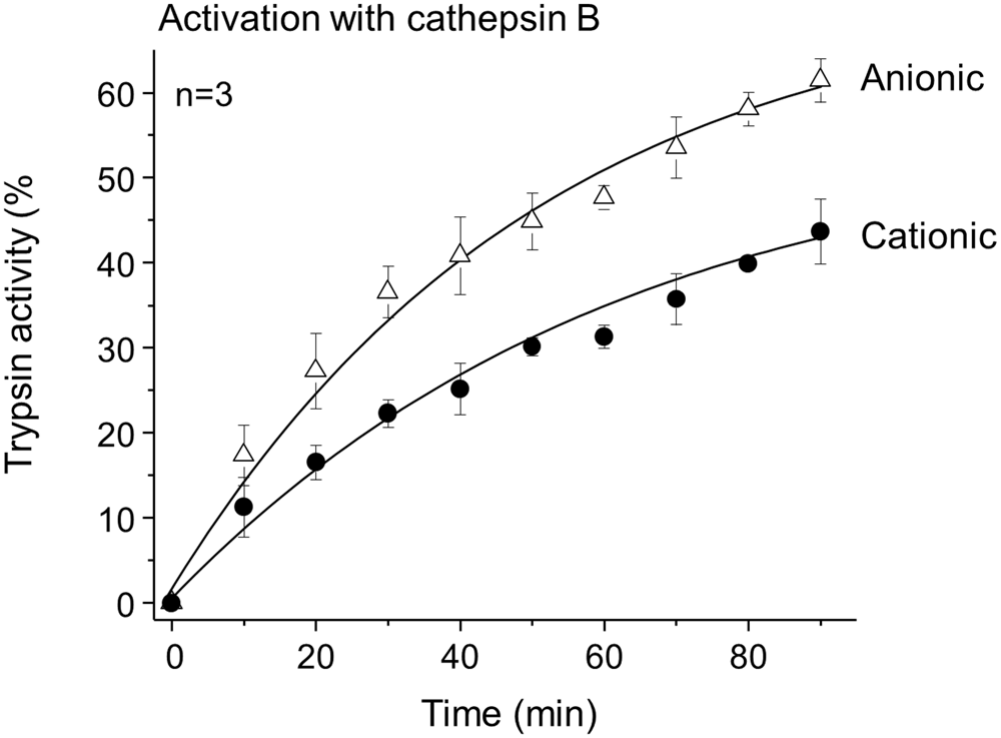
Activation of ferret trypsinogens by human cathepsin B. Anionic and cationic ferret trypsinogens were activated at 1 µM concentration with 8 µg/mL human cathepsin B at 37 °C in 0.1 M sodium acetate buffer (pH 4.0), 1 mM K-EDTA and 0.05% Tween 20, in 100 µL final volume. Aliquots (2 µL) were withdrawn at indicated times and trypsin activity was measured. Activity was expressed as percent of the potential maximal activity, which was determined in separate reactions by activation with enteropeptidase. Mean values with S.D. are shown.

Both ferret trypsinogen isoforms were readily activated by cathepsin B, the lysosomal cysteine protease responsible for intra-pancreatic trypsin activation in secretagogue-induced models of experimental acute pancreatitis (Fig. 6). This observation indicates that in the absence of trypsinogen autoactivation, cathepsin B-mediated trypsinogen activation may still contribute to pancreatitis development in ferrets.

## Discussion

In the present study, we purified the trypsinogen isoforms from the ferret pancreas and cloned their corresponding cDNAs. In agreement with genomic predictions, we identified three isoforms, two of which were expressed abundantly. In keeping with biochemical tradition, based on their isoelectric points, we named these isoforms anionic and cationic trypsinogen. The two ferret trypsinogens are 76% identical with each other and they exhibit a high degree of identity (~90%) with their corresponding anionic and cationic trypsinogen paralogs from the dog (*Canis lupus familiaris*) and the cat (*Felis catus*). Identity with the human namesakes is slightly lower; 83% for ferret anionic trypsinogen versus human anionic trypsinogen (PRSS2) and 72% for ferret cationic trypsinogen versus human cationic trypsinogen (PRSS1).

Comparative functional analysis of ferret trypsinogens and human cationic trypsinogen revealed normal activation with enteropeptidase but defective autoactivation in 1 mM calcium, which models conditions in the secretory pathway. These observations suggest that intra-pancreatic trypsinogen activation due to autoactivation is an unlikely event in the ferret pancreas and may not pose the same risk for pancreatitis as in humans. Cationic trypsinogen was strikingly defective in autoactivation even in the presence of high calcium concentrations, while anionic trypsinogen readily autoactivated under these conditions. The guinea pig trypsinogen, which is also cationic in nature, exhibited a similarly impaired autoactivation in our previous studies^6^. Both ferret trypsins were highly active on various peptide and protein substrates; indicating that poor autoactivation is not due to a catalytic defect. Anionic trypsin exhibited higher activity on a small peptide substrate and in the chymotrypsinogen activation assay than cationic trypsin, while both trypsins digested casein comparably. As observed with the guinea pig trypsinogen, ferret trypsinogens were readily activated by cathepsin B.

Inspection of the ferret trypsinogen sequences reveals some interesting differences from their human paralogs. The human regulatory nick sites for CTRC are absent; Leu81 is replaced with Asn in ferret anionic trypsinogen and Ser in ferret cationic trypsinogen, and Phe18 is replaced with Thr and Ile, respectively. These differences indicate that autoactivation of ferret trypsinogen is probably not regulated by chymotrypsins. The absence of this protective mechanism in the ferret pancreas is consistent with the weak ability of ferret trypsinogens to autoactivate, which poses no pathogenic risk. On the other hand, Arg122 is conserved in both ferret trypsinogens indicating that trypsin-mediated trypsinogen degradation and/or trypsin autolysis may occur in the ferret gut, presumably to re-cycle intestinally secreted trypsinogen protein.

The activation peptide of mammalian trypsinogens contains a characteristic tetra-aspartate sequence, which has been viewed as an enteropeptidase recognition motif, even though we found that mutation of these residues to alanine did not affect activation of human cationic trypsinogen by enteropeptidase^14^. The activation peptide Asp residues, however, strongly inhibit trypsinogen autoactivation and thereby serve as a protective biochemical mechanism against premature intra-pancreatic trypsinogen activation. Interestingly, in the ferret anionic trypsinogen Asp20 is replaced with a Glu residue, which is rarely found in trypsinogen activation peptides. We previously mutated all Asp residues individually to Glu in human cationic trypsinogen and found that mutation of Asp20 to Glu had a small inhibitory effect on autoactivation^14^. It is unlikely that Glu20 was evolutionarily selected in the ferret anionic trypsinogen to further suppress the otherwise slow intra-pancreatic autoactivation. It seems more reasonable to assume that Glu20 modifies the calcium binding affinity of the activation peptide in such a manner that results in optimal trypsinogen activation under the conditions that prevail in the ferret gut.

Human trypsinogens become post-translationally sulfated on Tyr154^15^. The functional significance of this modification is unclear and the presence of a common African polymorphism that abolishes trypsinogen sulfation suggests that it is dispensable for normal physiology^16^. Interestingly, Tyr154 is conserved in ferret anionic and cationic trypsinogens along with a flanking negatively charged residue (Glu156 and Asp156, respectively), suggesting that ferret trypsinogens might be sulfated. However, previously we found in human trypsinogens that Asp153 is an obligatory residue for efficient sulfation^16^ and this position contains an Asn and an Arg in the anionic and cationic ferret trypsinogens, respectively. Therefore, similarly to most other mammalian trypsinogens, the ferret trypsinogens are unlikely to undergo tyrosine sulfation.

We undertook the present studies to investigate whether properties of ferret trypsinogens are similar to those of human trypsinogens and whether the ferret may serve as a potential animal model to study human hereditary pancreatitis associated with trypsinogen mutations. We were encouraged by recent observations indicating that CFTR-deleted ferrets developed dramatic pancreatic disease that mimicked the human condition^8,9^. We found that similarly to humans, ferrets also express anionic and cationic trypsinogen isoforms to high levels with a slight preponderance of cationic trypsinogen. In contrast to human trypsinogens, ferret trypsinogens proved to be defective in autoactivation under conditions, which likely prevail at the site of pathological trypsinogen activation in the pancreas. Therefore, we conclude, ferrets are not a good model for human hereditary pancreatitis driven by trypsinogen autoactivation. However, the ability of cathepsin B to activate ferret trypsinogens indicates that intra-pancreatic trypsinogen activation may occur in ferrets, which can contribute to pancreatitis development even in the absence of trypsinogen autoactivation.

## Electronic supplementary material

Supplementary files

## Data Availability

Materials, data and protocols associated with this paper are available upon request.
