# Supplementary material for: Trypsinogen isoforms in the ferret pancreas
Source: Sci Rep. 2018 Oct 10;8:15094. doi: 10.1038/s41598-018-33423-w (PMC6180083; doi:10.1038/s41598-018-33423-w)
Supplement: Supplementary file 1 — Supplementary files [file 41598_2018_33423_MOESM1_ESM.pdf]

Trypsinogen isoforms in the ferret pancreas

Eszter Hegyi and Miklós Sahin-Tóth

Center for Exocrine Disorders, Department of Molecular and Cell Biology, Boston University Henry M. Goldman School of Dental Medicine, Boston, MA 02118

Supplementary Figure S1. Complementary DNA and deduced amino-acid sequence of the ferret anionic pre-trypsinogen. Amino-acid numbering starts with the initiator methionine codon. The activation peptide is highlighted in blue, Arg122 in red and the catalytic triad in yellow. The position of the gene-specific primers used for PCR amplification is underlined.

```
5'UTR
ACACTTCTGCCATC

1
ATG AAT CCA CTC CTG ATT CTT GCT TTT GTG GGA GCT GCT GTT GCT TTC CCC ACT GAT GAG
Met Asn Pro Leu Leu Ile Leu Ala Phe Val Gly Ala Ala Val Ala Phe Pro Thr Asp Glu
                                29
GAT GAC AAG ATC GTC GGT GGC TAC ACC TGC GAG GAG CAT TCC ATC CCC TAC CAG GTG TCC
Asp Asp Lys Ile Val Gly Gly Tyr Thr Cys Glu Glu His Ser Ile Pro Tyr Gln Val Ser

CTG AAC TCG GGC TAC CAC TTC TGC GGC GGC TCC CTC ATC AGT GAC CAG TGG GTG GTG TCC
Leu Asn Ser Gly Tyr His Phe Cys Gly Gly Ser Leu Ile Ser Asp Gln Trp Val Val Ser
        63
GCA GCT CAC TGC TAC AAG TCC CGC ATC CAG GTG AGG CTC GGA GAG CAC AAC ATC GAG GTC
Ala Ala His Cys Tyr Lys Ser Arg Ile Gln Val Arg Leu Gly Glu His Asn Ile Glu Val
81
AAT GAG GGG AAT GAG CAG TTC ATC AAC TCG GCC AAG GTC ATC CGC CAC CCC AAA TAT AGC
Asn Glu Gly Asn Glu Gln Phe Ile Asn Ser Ala Lys Val Ile Arg His Pro Lys Tyr Ser
                        107
AGC TGG TCC CTG GAC AAT GAC ATC ATG CTC ATC AAG CTG TCC TCG CCT GCT GTC CTC AAT
Ser Trp Ser Leu Asp Asn Asp Ile Met Leu Ile Lys Leu Ser Ser Pro Ala Val Leu Asn
    122
GCA CGC GTG TCC ACC ATA TCT CTG CCC TCC GCC TGT GCA GCC GCT GGC ACC CAG TGC CTC
Ala Arg Val Ser Thr Ile Ser Leu Pro Ser Ala Cys Ala Ala Ala Gly Thr Gln Cys Leu
                                                    154
ATC TCC GGC TGG GGC AAC ACC CTG AGC TCT GGC ATC AAC TAC CCT GAG CTG CTG CAG TGC
Ile Ser Gly Trp Gly Asn Thr Leu Ser Ser Gly Ile Asn Tyr Pro Glu Leu Leu Gln Cys

CTG GAC GCC CCA CTC CTG AGT CAG GCC CAG TGC GAA GCC TCC TAC CCC GGC CAG ATC ACG
Leu Asp Ala Pro Leu Leu Ser Gln Ala Gln Cys Glu Ala Ser Tyr Pro Gly Gln Ile Thr
                                                                            200
GAG AAC ATG GTT TGC GCT GGC TTC CTC GAG GGA GGC AAG GAC TCC TGC CAG GGT GAC TCT
Glu Asn Met Val Cys Ala Gly Phe Leu Glu Gly Gly Lys Asp Ser Cys Gln Gly Asp Ser

GGT GGC CCT GTG GTC TGC AAT GGA GAG CTC CAG GGC ATT GTC TCC TGG GGC TAT GGC TGT
Gly Gly Pro Val Val Cys Asn Gly Glu Leu Gln Gly Ile Val Ser Trp Gly Tyr Gly Cys

GCC CAG AAG AAC AAA CCT GGA GTT TAC ACC AAG GTG TGC AAC TTT GTA GAC TGG ATT AAG
Ala Gln Lys Asn Lys Pro Gly Val Tyr Thr Lys Val Cys Asn Phe Val Asp Trp Ile Lys
                        247
ACG ACC ATA GCT GCC AAC AGC TAA
Thr Thr Ile Ala Ala Asn Ser TER

3' UTR
AGCCCCCCCAGTCCTTCTGCCATCACTATGCTAATTAAATGTTCCTGTTAC
```

Supplementary Figure S2. Complementary DNA and deduced amino-acid sequence of the ferret cationic pre-trypsinogen. Amino-acid numbering starts with the initiator methionine codon. The activation peptide is highlighted in blue, Arg122 in red and the catalytic triad in yellow. The position of the gene-specific primers used for PCR amplification is underlined. The linked allelic variants c.123T>G (p.L41=) and c.*35C>T are shown in green.

```
5'UTR
ACATTCCTCAGTCAAATTCCAGGGAGCAACC

1
ATG AAG ACC TTC ATC TTC CTT GCC CTG CTG GGA GCT GCT GCT GCT TTC CCC ATT GAT GAC
Met Lys Thr Phe Ile Phe Leu Ala Leu Leu Gly Ala Ala Ala Ala Phe Pro Ile Asp Asp
                                29
GAT GAC AAG ATC GTT GGG GGC TAC ACC TGT CAG AGG AAT TCT GTT CCC TAC CAG GTG TCC
Asp Asp Lys Ile Val Gly Gly Tyr Thr Cys Gln Arg Asn Ser Val Pro Tyr Gln Val Ser
  G
CTT AAC TCG GGC TAT CAC TTC TGT GGT GGA TCC CTC ATC AAT TCC CAG TGG GTG GTG TCT
Leu Asn Ser Gly Tyr His Phe Cys Gly Gly Ser Leu Ile Asn Ser Gln Trp Val Val Ser
        63
GCA GCT CAC TGC TAC AAG TCC CGA ATC CAG GTG CGT CTG GGA GAA CAC AAC ATC GCA GTC
Ala Ala His Cys Tyr Lys Ser Arg Ile Gln Val Arg Leu Gly Glu His Asn Ile Ala Val
81
TCT GAG GGT GGT GAG CAA TTC ATC AAT TCA GCC AAG ATC ATC CGC CAC CCC AGA TAC AAC
Ser Glu Gly Gly Glu Gln Phe Ile Asn Ser Ala Lys Ile Ile Arg His Pro Arg Tyr Asn
                        107
CAA AAC ACT ATG GAT AAT GAC ATC ATG CTG ATT AAA CTG AGC TCT CCC GCC ACC CTC AAC
Gln Asn Thr Met Asp Asn Asp Ile Met Leu Ile Lys Leu Ser Ser Pro Ala Thr Leu Asn
    122
TCT CGA GTG TCT TCT ATC TCT CTA CCA AAA TCC TGT GCA GCT GCT GGT ACC CAG TGC CTC
Ser Arg Val Ser Ser Ile Ser Leu Pro Lys Ser Cys Ala Ala Ala Gly Thr Gln Cys Leu
                                                154
ATC TCT GGC TGG GGG AAC ACC CTG AGT ACT GGG CAA AGG TAT CCT GAT GTC CTG CAG TGT
Ile Ser Gly Trp Gly Asn Thr Leu Ser Thr Gly Gln Arg Tyr Pro Asp Val Leu Gln Cys

CTT CAA GCT CCC ATC CTC TCT GAC AGC ACT TGC CGC AAT GCC TAT CCT GGT CAG ATC AGC
Leu Gln Ala Pro Ile Leu Ser Asp Ser Thr Cys Arg Asn Ala Tyr Pro Gly Gln Ile Ser
                                                                            200
AGC AAC ATG ATC TGT CTG GGC TAC ATG CAG GGT GGA AAG GAC TCT TGC CAG GGT GAC TCT
Ser Asn Met Ile Cys Leu Gly Tyr Met Gln Gly Gly Lys Asp Ser Cys Gln Gly Asp Ser

GGT GGC CCT GTG GTC TGC AAC AGA GAG CTC CAG GGC ATT GTC TCC TGG GGT ATT GGC TGT
Gly Gly Pro Val Val Cys Asn Arg Glu Leu Gln Gly Ile Val Ser Trp Gly Ile Gly Cys

GCT CAA AAG GGC AAA CCT GGT GTC TAC ACC AAG GTC TGC AAC TAC GTG AGC TGG ATT CGG
Ala Gln Lys Gly Lys Pro Gly Val Tyr Thr Lys Val Cys Asn Tyr Val Ser Trp Ile Arg
                    246
CAA ACC ATT TCT GCC AAC TAA
Gln Thr Ile Ser Ala Asn TER

3' UTR                              T
GCATGTAAGGATGTGTATTGCTCTATGCACCATTCTCTTGGTCAATTTCACTTCCAACCATGCCTAAAACAGTATCTAAA
TAAAAACGTTTATTCCAC
```

Supplementary Figure S3. Complementary DNA and deduced amino-acid sequence of the ferret minor anionic pre-trypsinogen. Amino-acid numbering starts with the initiator methionine codon. The activation peptide is highlighted in blue, Arg122 in red and the catalytic triad in yellow. The position of the gene-specific primers used for PCR amplification is underlined. The allelic variant c.37G>A (p.A13T) is shown in green.

```
5'UTR
ACACTCCCCAGTCCAGTTCTAGGGAGCAGCC
1                                               A
ATG AAG ACC TTC ATC TTC CTT GCC CTG CTG GGA GCC GCT GTT GCT GTC CCC ATT GAG GAT
Met Lys Thr Phe Ile Phe Leu Ala Leu Leu Gly Ala Ala Val Ala Val Pro Ile Glu Asp
                                    29
GAT GAC AAG ATC GTT GGG GGC TAC ACC TGT CAG AAG AAT TCT GTT CCC TAC CAG GTG TTC
Asp Asp Lys Ile Val Gly Gly Tyr Thr Cys Gln Lys Asn Ser Val Pro Tyr Gln Val Phe

CTG AAC TTG GGC TAC CTC TTC TGT GGT GGC TCC CTC ATC AAT CCC CAG TGG GTG GTG TCC
Leu Asn Leu Gly Tyr Leu Phe Cys Gly Gly Ser Leu Ile Asn Pro Gln Trp Val Val Ser
        63
GCA GCT CAC TGC TAC AGG CCC CAA ATC CAG GTG CAT CTG GGA GAC CAC AAC ATC GCA ATT
Ala Ala His Cys Tyr Arg Pro Gln Ile Gln Val His Leu Gly Asp His Asn Ile Ala Ile
81
GTT GAG GGT GAT GAG CAA TTC ATC AAT TCA GCC AAG GTC ATC CGC CAC CCC AGA TAC AAT
Val Glu Gly Asp Glu Gln Phe Ile Asn Ser Ala Lys Val Ile Arg His Pro Arg Tyr Asn
                        107
GAA CAA AAC TTT GAT AAT GAC ATC ATG CTG ATT AAA CTG AGC TCA CCT GCC ACC CTC AAC
Glu Gln Asn Phe Asp Asn Asp Ile Met Leu Ile Lys Leu Ser Ser Pro Ala Thr Leu Asn
    122
TCC CGT GTG TCT ACT ATC TCT CTG CCA AAA TCC TGT GCG GAT GTT GAT ACC CAG TGC CTC
Ser Arg Val Ser Thr Ile Ser Leu Pro Lys Ser Cys Ala Asp Val Asp Thr Gln Cys Leu
                                                    154
ATC TCT GGC TGG GGG AAT ACC TGG AGT TTT GGG GAA AAT TTT CCT GAT GAC CTG CAG TGT
Ile Ser Gly Trp Gly Asn Thr Trp Ser Phe Gly Glu Asn Phe Pro Asp Asp Leu Gln Cys

CTT CAA GCT CCC ATC CTC TCT GAC AGC ACT TGC CGC GAT GCC TTC CCT GGG AGG ATC AGC
Leu Gln Ala Pro Ile Leu Ser Asp Ser Thr Cys Arg Asp Ala Phe Pro Gly Arg Ile Ser
                                                                            200
AGC AAC ATG ATC TGT CTG GGC TAC ATG CAG GGT AGA AAG GAT GCT TGC CAG GGT GAC TCT
Ser Asn Met Ile Cys Leu Gly Tyr Met Gln Gly Arg Lys Asp Ala Cys Gln Gly Asp Ser

GGT GGC CCT GTG GTC TGC AAC GGA GAG CTC CAG GGC ATT GTC TCC TGG GGC ATT GGC TGT
Gly Gly Pro Val Val Cys Asn Gly Glu Leu Gln Gly Ile Val Ser Trp Gly Ile Gly Cys

GCT CTA AAA GGC AAA CCT GGT GTC TAC ACC AAG GTC TGC AAC TAC GTG AGC TGG ATT CAG
Ala Leu Lys Gly Lys Pro Gly Val Tyr Thr Lys Val Cys Asn Tyr Val Ser Trp Ile Gln
                        246
GAG ACC ATT GCT GCC AAC TAA
Glu Thr Ile Ala Ala Asn TER

3' UTR
GCATGTAAGCACGTGTACTGCTCTATGCACCATTCTCTTGGACAATTTCACTTCCAACGATGCCTGAAACTGTATCTAAA
ATAAAAATGTATATTCCACATC
```
